# Reply to “Ten Simple Rules for Getting Published”

**DOI:** 10.1371/journal.pcbi.0030190

**Published:** 2007-09-28

**Authors:** Eric Grosch

Rule 10 for getting published [1] carries advice to publish in journals of high impact (high citation rate). Riding the coat-tails of eminent, high-impact journals is good marketing, but the task is easier said than done, because the higher the impact is the greater is the competition for print space and the more likely the editor is to offer unhelpful feedback, such as a statement on a form letter that he rejects many worthwhile manuscripts for lack of space. Good science may appear in the pages of journals of many degrees of impact. In support of that notion, current impact factors [2] appear in Table 1 for each of the journals (or successor—Am J Epidemiol continued J Chron Dis) cited in this essay (see References).

**Table 1:**
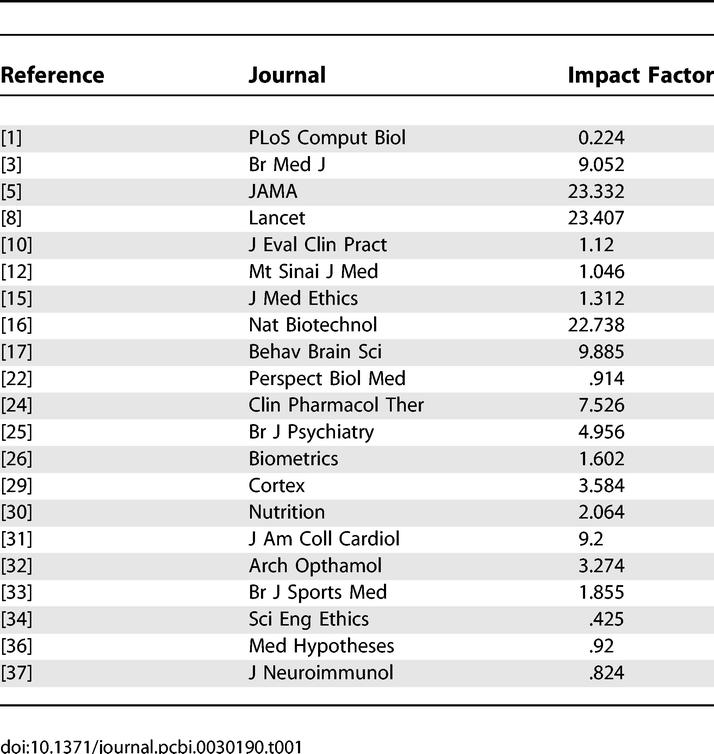
Impact Factors for Journals Referenced in This Essay

Yet, a journal's high eminence and high impact may bespeak its rigid orthodoxy, rather than its high quality. Rule 10 may hold for journals, such as *PLoS Computational Biology,* in which objective science, evidence, and the GIGO (“garbage in, garbage out”) principle count for something. Eminence-based medicine [3,4] too often substitutes—and poorly—for evidence-based medicine [5]. Altman deplored poor medical research [6], which too often appears in high-impact medical journals, and suggested, “incorrect procedures . . . can be hard to stop . . . from spreading . . . like a genetic mutation” [7]. Consensus in medicine [8] too often permits false doctrine to masquerade as “standard of care,” just as an ad blitz may build a public consensus on specious claims that favor sale of a certain brand of snow tire [9]. Medical science and its “opinion-leaders” were arguably tardy in complying with Rule 6, good science [1], in recognizing Helicobacter pylori in peptic ulcer disease [10,11], thrombolytic therapy for myocardial infarction [4,12], questioning post-menopausal estrogen [10,13], and preventing thousands of crib deaths by rejecting Benjamin Spock's high-impact advice to lay babies prone [14], among other instances [15].

In medical journals, eminence-based medicine [3,4] predominates, and censorship by editors, in attempts to save face, may impair the vitality [16] and self-correction [17,18] of science and the protection of “the literature and the reader from identifiable error” [19], despite editorial lip service to “evidence-based medicine” [5].

Helpful first steps to remedy the current malaise might consist of prompting editors of scientific journals, of all levels of impact, to improve peer review by encouraging substantive dialogue [20], by adhering to logic [21,22] and to valid statistical inference [23–25], by encouraging authors to provide readers access to raw data [7,26–31], the better that readers might verify or challenge published conclusions, by issuing to editorial peer reviewers a “plea for rigor” [32] and diligence [33] by requesting them to “state the rationale, and present the evidence, for exceptions taken to the manuscript” [32], and by incorporating the dialectical scientific brief [34], rather than by perpetuating current inequities: a) for each hour put in by a journal reviewer or editor, the author puts in about seven hours... [35]; b) the average time spent reviewing a paper is less than two hours in medicine [36]; c) the editor invariably defends the reviewer's call. After all, who are we to question the decision of someone who may have devoted much time to the manuscript [37]?

High-impact medical journals too often nurture sacred cows by taking in and putting out orthodox garbage and rejecting innovative pearls. Then the Institute of Medicine wonders why 44,000 to 98,000 patients per annum die of preventable medical errors in the hospitals of the United States [38].
